# Phase Change Material for Thermotherapy of Buruli Ulcer: A Prospective Observational Single Centre Proof-of-Principle Trial

**DOI:** 10.1371/journal.pntd.0000380

**Published:** 2009-02-17

**Authors:** Thomas Junghanss, Alphonse Um Boock, Moritz Vogel, Daniela Schuette, Helmut Weinlaeder, Gerd Pluschke

**Affiliations:** 1 Section Clinical Tropical Medicine, University Hospital, Heidelberg, Germany; 2 Leprosy Relief Emmaus-Switzerland (ALES), Bureau Régional pour l'Afrique, Yaoundé, Cameroon; 3 Department of Medical Parasitology/Infection Biology, Swiss Tropical Institute, Basle, Switzerland; 4 Bavarian Center for Applied Energy Research, Functional Materials for Energy Technology, Wuerzburg, Germany; Sabin Vaccine Institute, United States of America

## Abstract

**Background:**

Buruli ulcer (BU) is an infection of the subcutaneous tissue leading to chronic necrotizing skin ulcers. The causative pathogen, *Mycobacterium ulcerans*, grows best at 30°C–33°C and not above 37°C. We explored the safety, tolerability and efficacy of phase change material (PCM), a novel heat application system for thermotherapy of BU.

**Methodology/Principal Findings:**

In a prospective observational single centre proof-of-principle trial in Ayos/Cameroon, six laboratory reconfirmed patients with ulcerative Buruli lesions received 28–31 (ulcers ≤2 cm) or 50–55 (ulcers >2 cm) days of thermotherapy with the PCM sodium acetate trihydrate as heat application system. This PCM is widely used in commercial pocket heat pads, it is easy to apply, rechargeable in hot water, non-toxic and non-hazardous to the environment. All patients enrolled in the trial completed treatment. Being completely mobile during the well-tolerated heat application, acceptability of the PCM bandages was very high. In patients with smaller ulcers, wounds healed completely without further intervention. Patients with large defects had skin grafting after successful heat treatment. Heat treatment was not associated with marked increases in local inflammation or the development of ectopic lymphoid tissue. One and a half years after completion of treatment, all patients are relapse-free.

**Conclusions/Significance:**

Our reusable PCM-based heat application device appears perfectly suited to treat BU in endemic countries with limited resources and infrastructure.

**Trial Registration:**

Controlled-Trials.com ISRCTN88392614

## Author Summary

Buruli ulcer is an infection of the subcutaneous tissue leading to chronic necrotizing skin ulcers. The causative pathogen, *Mycobacterium ulcerans*, grows best at 30°C–33°C and not above 37°C, and this property makes the application of heat a treatment option. We achieved a breakthrough in heat treatment of Buruli ulcer by employing the phase change material sodium acetate trihydrate as a heat application system for thermotherapy, which is widely used in commercial pocket heat pads. It is easy to apply, rechargeable in hot water, non-toxic and non-hazardous to the environment. Six laboratory reconfirmed patients with ulcerative Buruli lesions were included in the proof-of-principle study and treated for four to six weeks. In patients with small ulcers, wounds healed completely without further intervention. Patients with large defects had skin grafting after successful heat treatment. Heat treatment was not associated with marked increases in local inflammation or the development of ectopic lymphoid tissue. One and a half years after completion of treatment, all patients are relapse-free. The reusable phase change material–based heat application device appears perfectly suited for use in remote Buruli ulcer–endemic areas of countries with limited resources and infrastructure.

## Introduction

Buruli ulcer (BU) is a chronic necrotizing disease of skin and soft tissue caused by *Mycobacterium ulcerans* [1]. The disease starts as a subcutaneous nodule, papule or plaque that eventually ulcerates and progresses over months to years. In BU lesions, clumps of extra-cellular acid-fast organisms surrounded by areas of necrosis are found primarily in subcutaneous fat tissue [2]. *M. ulcerans* produces a macrolide toxin, mycolactone, which is associated with tissue destruction and local immunosuppression [3]. BU has been reported in >30 countries, but the major burden lies on children living in remote areas of West Africa associated with swamps and stagnant water bodies. Traditionally wide excision of the infected tissue alone was the standard treatment for BU. This is hampered by traumatic interventions, high cost and very high recurrence rates [4]. Chemotherapy with streptomycin and rifampicin is currently re-evaluated as an adjunct treatment to surgery and as a therapy in its own right [5],[6],[7],[8].

*M. ulcerans* differs from most other pathogenic mycobacteria in that it grows best at 30–33°C and not above 37°C [9]. This characteristic feature of the pathogen was first used for therapeutic purposes in the early 1970s. Meyers et al. treated 8 patients from Zaire maintaining a temperature of approximately 40°C in the ulcerated area for a mean duration of 68 days [10]. There was no evidence of local recurrence during follow-up periods of up to 22 months. Based on this impressive success rate, WHO guidelines listed the application of heat as a treatment option for BU [11]. However, the heat application devices employed so far were impractical in most endemic countries. Here we describe the use of a cheap and easy to apply phase change material (PCM) device suitable for thermotherapy of BU in countries with limited resources.

## Methods

### Study participants

#### Eligibility criteria for participants and case definition

All patients between 6 and 30 years of age with an ulcer at the lower or upper arm or leg with a diameter of up to 12 cm suggestive for BU on clinical grounds in the catchment area of the Buruli treatment center Ayos/Cameroon were candidates for inclusion in the study. They were not admitted to the study if any of the following criteria were present: (1) clinical signs and symptoms of communicable diseases other than BU (fever, weight loss, night sweats, persistent cough, jaundice, pulmonary or myocardial dysfunction, CNS involvement, ascites, pleural effusion), (2) clinical signs and symptoms of non-communicable diseases (myocardial, pulmonary, renal, CNS) and (3) inability to confirm BU using laboratory methods.

A BU case was defined as a patient with an ulcer diagnosed as BU on clinical grounds and positive results in at least two of the three laboratory tests (PCR, detection of AFB on microscopy and histopathology) performed.

#### Laboratory confirmation of clinical diagnosis

On day 0 four swabs from the undermined edges and one diagnostic biopsy were taken from all patients enrolled into the trial on clinical grounds. A second set of biopsies was taken in week 4 of thermotherapy to assess histopathological changes in response to heat treatment. All samples were investigated by microscopy for acid-fast bacilli (AFB) after Ziehl Neelsen (ZN) staining and by IS2404 real-time PCR [12]. Histopathological changes typical for BU [12] were recorded in the initial biopsies and the follow-up biopsies in week 4 of thermotherapy.

Immediately after performing the punch biopsies, tissue samples were fixed in 4% neutral-buffered PFA (paraformaldehyde) for 24 h and subsequently transferred to 70% ethanol for short term storage and transport. Biopsies were dehydrated, embedded in paraffin, cut into 5 µm thin sections and retrieved on glass slides. After dewaxing and rehydration, sections were stained with haematoxylin/eosin (HE) and ZN. Immunohistochemistry (IHC) was performed with antibodies against Elastase (polymorphonuclear neutrophils [PMNs]; Dako) and CD3 (T lymphocytes; Dako). Staining was performed using Vector NovaRED and haematoxylin.

#### The setting and location where the data were collected

Volunteers were recruited in the catchment area of the Buruli treatment center at the hospital Ayos/Cameroon, identified by active and passive case detection. The treatment center has a longstanding collaboration with and is supported by Leprosy Relief Emmaus-Switzerland (ALES). It maintains a very well equipped and functioning operation theatre, wards for pre- and postsurgical care, physiotherapy and a school which is of importance because the majority of patients with this disease are children and convalescence after excision of ulcers and skin grafting takes many months in the majority of patients. Dr. A. Um Boock, the director of the ALES Bureau Régional pour l'Afrique, and his team are very experienced in the diagnosis and management of patients with Buruli ulcer, including surgery and skin grafting.

#### Ethical approval and informed consent

The protocol was approved by the National Ethics Committee of Cameroon and the Ethics Committee of the University Hospital, Heidelberg, Germany. Patients were enrolled in the study only after informed written consent was obtained from them or their care providers.

### Interventions

#### Heat application

**Figure 1:**
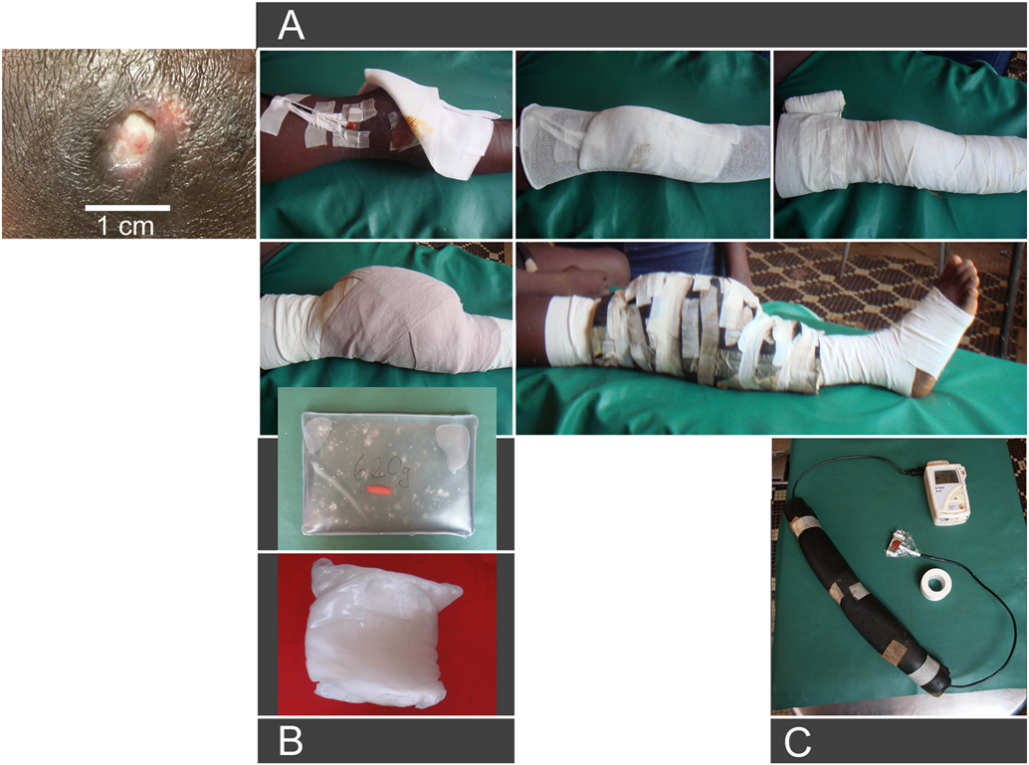
Mounting of the PCM-based heat application system and temperature monitoring device. (A) PCM pack and bandage mounted for treating an ulcer on the lower limb (patient 2) and temperature monitoring system, (B) PCM pack with sodium acetate trihydrate in the fluid phase before initiating the crystallisation process with the starter (red), sodium acetate trihydrate in the solid phase after the stored heat has been discharged, (C) temperature monitoring system with the sensor connected to the data logger to record the temperature at the skin surface as part of the clinical trial documentation. This will not be needed when the device is put into routine use.

Commercially available plastic bags filled with the PCM sodium acetate trihydrate were used. Starters were placed in the bags to initiate the crystallisation process (Fig. 1). Size of filled bags is 21 cm×15 cm×2 cm with an average weight of 800 g. The melting temperature of the PCM sodium acetate trihydrate is 58°C. The unique feature of PCM is its thermal energy storing capacity combined with an almost constant temperature during the liquid-solid phase transition. This property is widely used in commercial pocket heat pads.

**Figure 2:**
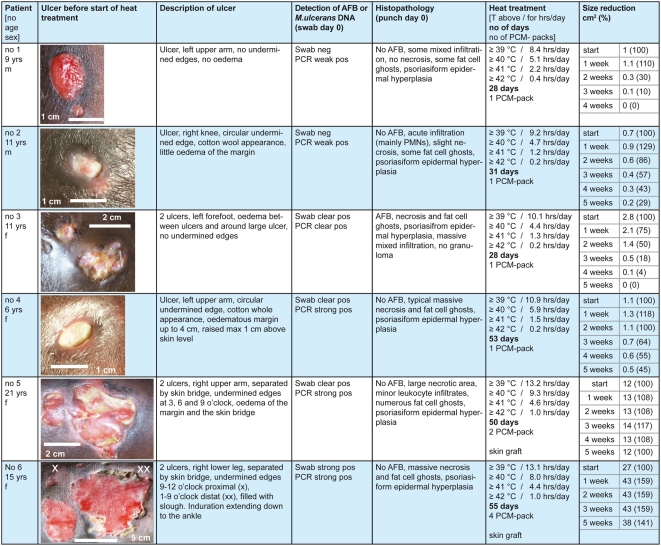
Baseline data, heat treatment schedules and results.

After cleaning and sterile dressing of the ulcers a heat sensor connected to a data logger (testo 177-T3, testo AG, Lenzkirch, Germany) was placed on healthy skin at the edge of the ulcer. The area of contact between skin and PCM packs was protected by tube gauze and a layer of elastic bandage to lower the PCM working temperature from 58°C to the therapeutic target temperature of 40°C at skin surface (Fig. 1). Temperatures of ≤58°C do not cause burns when not applied for prolonged periods of time. Skin temperatures of up to 43°C were accepted and well tolerated for short intervals of time immediately after mounting the PCM bandage. The affected skin (ulcer /oedema/induration) plus a safety margin of several centimeters was covered by one to four PCM packs per session depending on the size of the total area to be treated (Fig. 2). The PCM packs were fixed with several layers of elastic bandage. A thermal insulation layer, commercially available to insulate hot water pipes, was used to reduce heat loss to the environment and to reinforce positioning of the PCM packs (Fig. 1). This allowed patients to move around freely. The 24 hours protocol was as follows: 8.00: Clinical progress assessment, cleaning and dressing of ulcers and renewal of PCM-packs, photo documentation at, on average, 3 day intervals. 12.00: Removal of PCM-packs, dressing of the wound to protect from contamination during a 5 hour pause of heat treatment, skin care with fatty cream. 17.00: Additional wound cleaning and dressing, if needed, renewal of PCM-packs. 22.00: Renewal of PCM-packs.

Clinical observations (appearance of the ulcer and the surrounding heat exposed skin, overall clinical assessment of the patient) were recorded daily at the above mentioned time points on case record forms (CRF). Temperature at the skin surface was automatically recorded at 10 minute intervals and stored in a small data logger carried by the patients. Temperature data were transferred daily to a notebook and checked for therapeutic and safety margins (testo software ComSoft 3.4, testo AG, Lenzkirch, Germany).

Patients with small ulcers and without significant oedema (patients 1, 2, 3) received heat treatment for 28–31 days, patients with large ulcers and/or significant oedema (patients 4, 5, 6) for 50–55 days.

### Study objectives

In the current study we tested the hypotheses that

1. PCM-based heat application is safe and comfortable for patients
2. with PCM based heat application the results of the thermotherapy study of Meyers et al [10] can be reproduced, i.e. primary healing of Buruli ulcer without relapse can be achieved

### Primary outcomes

1. Proportion of patients completing 28–31 days of heat treatment in patients with small ulcers (≤2 cm) or 50–55 days in patients with large ulcers (>2 cm) and ulcers with prominent surrounding oedema
2. Proportion of patients cured 6 months after completing heat treatment (including skin grafting where necessary). Cure is defined as complete closure of the wound by epithelialisation or scarification or by skin graft.
3. Proportion of patients who are recurrence free 18 months after completing heat treatment

### Secondary endpoint

Histopathological responses in week 4 of thermotherapy compared to reference samples at day 0.

## Results

### Participant flow

Seven patients with ulcers suggestive for BU on clinical grounds were recruited by active and passive case detection. In six of the seven patients enrolled the diagnosis was laboratory confirmed.

### Protocol deviations

We extended the total duration of heat application of large ulcers (>2 cm) and ulcers with prominent surrounding oedema from 4 week to 50–55 days and did not, as originally planned, treat small and large ulcers equally for 4 weeks only. This was done even though all ulcers appeared clinically healed after 4 weeks of heat treatment, independent of size and surrounding oedema. This decision was taken on the basis of the results of the punch biopsies in week 4 of thermotherapy showing residual AFB with intact rod-shaped appearance.

### Recruitment and follow-up

Eligible patients were recruited between February 28, 2007 and March 3, 2007. Patients stayed in the hospital during the course of heat treatment and thereafter until the wound was closed (patients with small ulcers; patients 1, 2, 3, 4,) or skin grafted (patients with large ulcers; patient 5 and 6). All patients were followed up until 18 months after completion of heat treatment.

### Baseline data

The age range of the seven patients enrolled was six to 21 years. Three patients had single ulcers on the upper and four had single ulcers on the lower extremities. Medical history and physical examination revealed no significant health problem other than BU. In six out of seven patients enrolled in the study on clinical grounds, diagnosis was laboratory confirmed. The unconfirmed patient was excluded from the analysis (Fig. 2).

### Outcomes

**Figure 3:**
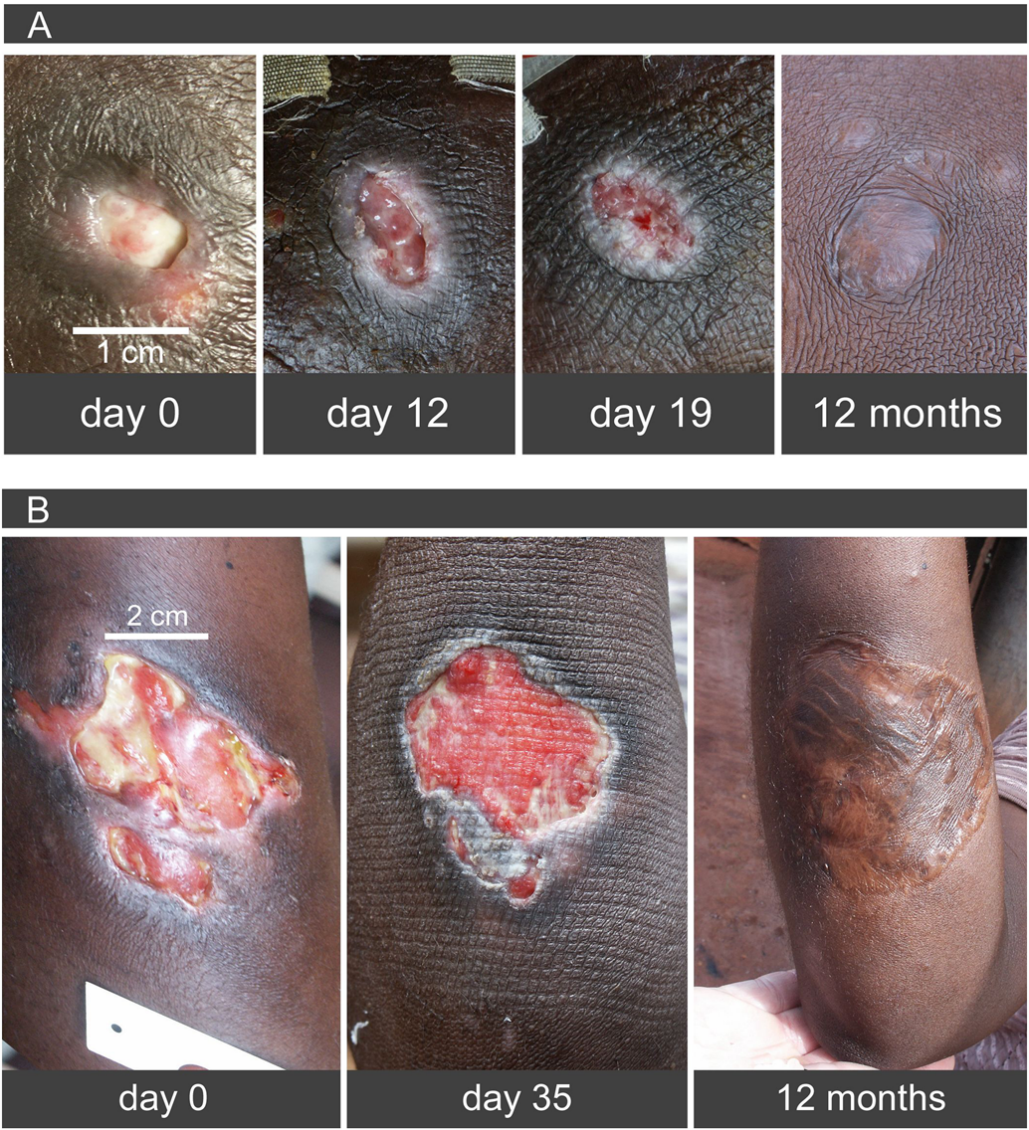
Healing of Buruli ulcers under PCM-based heat treatment and long term results. (A) Patient 2, (B) patient 5: Progress of healing during heat treatment. Note in particular early onset of epithelialisation. Far right follow-up 12 months after completion of heat treatment. Patient 5 (B) after skin grafting.

All patients enrolled into the trial completed treatment. In all patients temperatures at the lesion and over a wide margin of healthy looking skin were maintained above ≥39°C for between 8.4 and 13.2 hours and ≥40°C for between 4.4 and 9.3 hours per day (Fig. 2). Undermined margins collapsed between day 1 and day 3. Epithelialization started in all patients between 4 and 11 days after the start and was almost completed in patients 1, 2, and 3 at the end of heat treatment (Fig. 2 and Fig. 3). In particular in patients with oedematous lesions (patients 4, 5) white discharge from ulcers was observed during initial treatment for various lengths of time. The two patients with large defects (patients 5 and 6) had skin grafting after completion of heat treatment (Fig. 3B).

All six reconfirmed patients were healed and relapse-free 18 months after completion of treatment.

In the punch biopsies taken prior to start of treatment, histopathological changes characteristic for BU, such as fat cell ghosts, deep dermal necrosis and/or psoriasiform epidermal hyperplasia, were found in six patients (Fig. 2). All patients yielded positive semi-quantitative IS2404 real-time PCR results. AFBs were detected in swabs or punch biopsies of 4 out of 6 patients included in the study.

Analysis of serial sections of punch biopsies taken at day 0 and in week 4 of thermotherapy showed, that heat treatment was not associated with marked increases in local inflammation, the development of ectopic lymphoid tissue or haemorrhages. At both time points small numbers of both polymorphonuclear cells as members of the innate and T cells as members of the adaptive immune system were present, with polymorphonuclear cells mainly located around necrotic areas and T cells more confined to areas close to vessels in the upper dermis. Only the lesion of patient 3 contained both on day 0 and in week 4 of thermotherapy mixed cellular infiltrates, which were much more pronounced than in typical untreated BU lesions.

### Safety and tolerability of PCM-based heat treatment, adverse events

The heat treatment procedure was very well tolerated by all patients. Patients with one (patients 1, 2, 3, 4) and with two PCM packs (patient 5) could move around freely and did not feel unacceptably disturbed during their daily activities nor during sleep at night. Patient 6 with four PCM packs also walked with acceptable restrictions and slept largely undisturbed. None of the patients and their guardians requested termination of treatment at any time. Temperatures between 40–43°C were observed only for short intervals of time immediately after mounting of the PCM packs without causing unacceptable discomfort. Only initially a few small blisters were occasionally observed. With a simple patient-controlled method the therapeutic target temperature of 40°C at skin surface was quickly reached and maintained without further side effects.

## Discussion

Successful treatment of BU with heat has been reported in individual patients and small case series since 1950 [10],[13],[14],[15]. This has not been carried further into clinical research and practice due to the fact that available heat application systems were cumbersome and not suited for use in developing countries. We achieved a break through by employing PCM packs as a cheap heat application system which is rechargeable in hot water, non-toxic and non-hazardous to the environment. In this proof-of-principle study we demonstrated that our heat application system is easy to use and allows the patient to move freely.

Family members and the hospital community accepted the treatment very well and favoured it over other treatments currently offered (surgery, antibiotics). Nurses quickly adopted the techniques of mounting the PCM packs and of recharging the packs in boiling water. The only side effects observed were sensation of excessive heat for a short period after applying the PCM packs. Lowering of the temperature at the skin surface by an elastic bandage interposed between tube gauze and PCM packs reliably prevented skin irritation and development of blisters, which may occur if the initial temperature at skin surface is less rigorously controlled.

With our PCM-based heat application system we reproduced the excellent results of the thermotherapy study of Meyers' group in 1974 [10] with significantly shorter heat application times both with respect to length of heat treatment per day (close to 24 hours [39°C–40.5°C] vs a mean of 10 hours, range 8.4–13.2 hours [≥39°C]) and to total heat application time (28 to 115 days vs 28 to 55 days). Since both systems worked at the same temperature range measured at skin surface, the minimum length of heat application to achieve healing of BU appears to be in the range of our heat treatment schedule or even shorter.

The initial clinical improvement of ulcerative lesions in our series was as fast as in the patient series of Meyers et al. As early as three days after initiation of heat treatment undermined ulcer margins collapsed and the skin attached to the underlying subcutaneous tissue with re-epithelialization starting at the edges. Discharge of the wound decreased over various lengths of time. Firm attachment of the affected skin was complete only after discharge stopped. By using heat treatment alone no viable tissue is lost and even the overarching margins at undercutting edges are often rescued. Lesions were clinically inactive in all of our patients with very good granulation and re-epitheliazation responses after 28 days of heat treatment. In one of our patients (patient 6) non-viable tissue extended far beyond the ulcerated area, which had to be excised before skin grafting. In this patient and one other patient with a large defect (patient 5) skin grafting was performed after a good granulation response had been achieved. Currently, all our patients are relapse-free 18 months after completion of heat therapy.

Rifampicin/streptomycin chemotherapy of BU is associated with the development of ectopic lymphoid tissue in the lesions [16]. In some patients, effects reminiscent of the immune reconstitution syndromes observed in tuberculosis and leprosy patients after highly active antiretroviral therapy [17] are observed. In contrast, heat treatment did not lead to massive increases in local inflammation and this less vigorous response may favour rapid re-epithelialization. Also haemorrhages, which are regarded as negative indicators for uncomplicated wound healing [18] were not observed.

Results of two pilot studies, the study of Meyers et al. in the 1970s [10] and our study, demonstrate that heat is a highly efficacious therapy for *M. ulcerans* disease. Use of PCM packs represents a break through for thermotherapy with respect to its practicality in endemic areas with poor infrastructure. Further optimization of the heat treatment schedule should make it suitable for community application.

## Supporting Information

Checklist S1 CONSORT checklist
(0.06 MB DOC)

Protocol S1 Study protocol “Phase change material to treat Buruli ulcer through heat treatment”
(0.09 MB DOC)
