# Supplementary material for: Phase Change Material for Thermotherapy of Buruli Ulcer: A Prospective Observational Single Centre Proof-of-Principle Trial
Source: PLoS Negl Trop Dis. 2009 Feb 17;3(2):e380. doi: 10.1371/journal.pntd.0000380 (PMC2637542; doi:10.1371/journal.pntd.0000380)
Supplement: Protocol S1 — Study protocol “Phase change material to treat Buruli ulcer through heat treatment” (0.09 MB DOC) [file pntd.0000380.s002.doc]

**Study protocol**

**“Phase change material to treat Buruli ulcer through heat treatment”**

**Project partners**

***Partner 1 (coordinating partner)***

Dr. Thomas Junghanss MD and

Dr Moritz Vogel MD

Section Clinical Tropical Medicine

University Hospital

INF 324

D - 69120 Heidelberg / Germany

phone +49 - 6221 – 56-3-4904

fax +49 - 6221 – 565204

[thomas.junghanss@urz.uni-heidelberg.de](mailto:thomas.junghanss@urz.uni-heidelberg.de)

***Partner 2***

Dr. Alphonse Um Boock MD

ALES

Bureau Régional pour l’Afrique

Yaoundé / Cameroon

phone: +237 222 23 78

fax: +237 222 05 63

[umboock@yahoo.fr](mailto:umbook@yahoo.fr)

***Partner 3***

Prof. Gerd Pluschke and

Daniela Schütte

Molecular Immunology Unit

Swiss Tropical Institute

Socinstr. 57

CH 4002 Basel / Switzerland

phone: + 41 61 2848235

fax: + 41 61 2848101

[Gerd.Pluschke@unibas.ch](mailto:Gerd.Pluschke@unibas.ch)

***Partner 4***

Dr.Helmut Weinläder

Bavarian Center for Applied Energy Research (ZAE Bayern)

Thermal Insulation and Heat Transfer

Am Hubland

D-97074 Würzburg / Germany

phone +49-931-7056448

fax +49-931-7056460

[helmut.weinlaeder@zae.uni-wuerzburg.de](mailto:helmut.weinlaeder@zae.uni-wuerzburg.de)

**Background and rational**

***Aim of the pilot trial***

To deliver in a clinical pilot study the “proof of principle” of the efficacy of heat treatment (Phase change material bandage = PCM - bandage) in patients with Buruli ulcers

***Rational***

PCM-based heat treatment is promising as an alternative or the least adjunct to current therapies available or under evaluation. Additionally and importantly it appears suitable for countries with limited resources because PCM bandages are cheap, easy to apply, easy to recharge in hot water or by solar heat and non-toxic and environmentally safe.

***Background***

Buruli ulcer is a devastating skin disease caused by *Mycobacterium ulcerans*, a pathogen belonging to the same family of organisms that cause tuberculosis and leprosy. Unlike these well known diseases, Buruli ulcer is a poorly understood disease that has emerged dramatically since the 1980s (Johnson et al., 2005). It is widespread in tropical and subtropical countries and cases have been identified in more than 30 countries in Africa, the Americas, Asia and the Western Pacific. West Africa is by far the most severely affected geographical area, with thousands of cases now being reported every year. The disease occurs most commonly in poor communities in remote rural areas. Around 70% of those infected are children under the age of 15 years. The causative agent has unique features and its exact mode of transmission is unknown. A remarkable feature of *M. ulcerans* disease is that although a single Buruli ulcer may contain millions of extracellularly growing bacteria (Rondini et al., 2003, 2005), the host does not mount an acute inflammatory response to the presence of the bacteria. A macrolide toxin, designated mycolactone, seems to play a key role in the local immunosuppression in Buruli ulcer. Treatment options for Buruli ulcer available today are unsatisfactory with wide surgical excision and skin grafting as the standard treatment over the past decades. The recurrence rates ranging between16 and 48% are unacceptably high. Antibiotics have been repeatedly evaluated with widely varying results. Recently Rifampicin / Streptomycin are again on trial focusing on their efficacy as primary and as adjunctive treatment combined with surgery (WHO 2004b).

***Heat treatment***

*M. ulcerans* differs from most other pathogenic mycobacteria in that it grows best at 30 - 33°C and not above 37°C. This property of *M. ulcerans* has first been used for therapeutic purposes in the early '70ies. Meyers et al. treated 8 patients from Zaire maintaining approximately 40ºC in the ulcerated area over a mean time of 68 days. There was no evidence of local recurrence during follow-up periods of up to 22 months (Meyers et al., 1974) contrasting to recurrence rates of up to 48 % after surgery (WHO 2001, Teelken et al., 2003). The success rates were impressing and WHO guidelines listed the application of heat as a treatment option for Buruli ulcer since then (WHO 2001). However, the heat application devices employed so far were impractical in most endemic countries being the roadblock for further trials and implementation of this highly effective method into the field. (Thangaraj et al 1999, van der Werf et al 1999).

In their evaluation of “Antimycobacterials for treating Buruli ulcer” the Cochran Skin Group recently came to the conclusion that heat therapy of Buruli ulcer needs further evaluation (<http://www.nottingham.ac.uk/~muzd/protocols/proto36AntiMyc.htm>).

**Study protocol**

**Study design**

Prospective observational study in 10 patients with single lesions of *M. ulcerans* ulcers treated with heat applied through phase change material (PCR) bandages

**Study site**

The study will take place in the hospital of Ayos / Cameroon. This hospital has a longstanding collaboration with and is supported by Aide aux Lépreux Emmaus-Suisse (ALES). With decreasing incidence and prevalence of leprosy in recent years Ayos hospital has moved into the field of Buruli disease. The hospital has become a regional treatment centre for patients with Buruli ulcers. The hospital maintains a very well equipped and functioning operation theatre, wards for pre- and postsurgical care, physiotherapy and a school (which is of importance because the majority of patients with this disease are children and reconvalescense after excision of ulcers and skin grafting takes in the majority of patients many months). Dr. A. Um Boock, the director of the ALES Bureau Régional pour l’Afrique, and his team are very experienced in the diagnosis and management of patients with Buruli ulcer, including surgery and skin grafting.

**Recruitment of patients**

10 Buruli ulcer patients will be recruited through passive and active case finding.

**Enrollment of patients**

Patient fulfilling the inclusion criteria (see section “inclusion criteria” below) will be fully informed about the clinical trial and written informed consent will be secured of the patients who agree to participate.

All patients who have consented will be submitted to a full assessment (see section “screening of patients” below and CRFs attached). Patients with exclusion criteria specified in the section “exclusion criteria” (see below) will be excluded. This procedure will be carried out until 10 patients are enrolled.

All enrolled patients will receive a booster immunization of diphtheria/tetanus/polio vaccine including those enrolled but not included into the trial.

**Inclusion criteria**

Patients (age 8 – 30 years) with a single ulcer (diameter 5 – 12 cm) at the upper or lower arm or leg clinically diagnosed as Buruli ulcer (WHO 2001).

**Exclusion criteria**

In view of the unproblematic nature of the treatment no relevant adverse - effects are foreseen. Nevertheless, patients with significant underlying other communicable and non-communicable diseases will be excluded in this first pilot study. With respect to communicable diseases these are in particular diseases presenting with clinical signs and symptoms of systemic involvement (fever, weight loss, night sweats, persistent cough, jaundice, pulmonary and myocardial dysfunction, CNS-involvement, ascites, pleural effusion), e.g. clinically overt HIV/AIDS, active hepatitis and other viral diseases, tuberculosis, leprosy. Patients excluded with clinically significant signs and symptoms of non-communicable diseases (myocardial, pulmonary, renal, CNS) include patients with heart failure, obstructive lung disease, kidney disease, cirrhosis of the liver, psychiatric diseases. We have designed the examination on enrollment accordingly to identify these patients and to exclude them from the study (see CRFs attached).

**Screening of patients to verify inclusion / exclusion criteria**

From each patient a full medical history will be taken and each patient will have a full physical examination (see CRFs).

**Clinical and laboratory diagnosis of lesion at enrollment**

At the time of enrolment the diagnosis of “Buruli ulcer” is clinical following the WHO guidelines (WHO 2002).

The ulcer will be measured (max. / min. diameters), the features of the ulcer will be described and particular attention will be paid to the undercutting edges. The ulcer will be photographed with an electronic camera (with a mm-scale in place) and the images will be stored electronically. Swabs from the undermined edges and a 0.4 mm punch biopsy will be taken on day 0 from each patient for laboratory confirmation of the clinical diagnosis according to the WHO recommendations. Additionally, a punch biopsy will be taken on day 21 and bacterial load will be analyzed in order to estimate the probability of relapse

**Treatment procedures (CRFs see annexe)**

1. The ulcer will be cleaned with an antiseptic daily. After cleaning the ulcer will be covered with polyvidon paste and sterile pads which will be fixed with a thin layer of a bandage (this will at the same time prevent discomfort at the skin due to direct contact of the PCM bandage with the skin; it will also fix a temperature sensor on the skin surface)
2. The standardized PCM-bandage will be wrapped around the part of the limb with the ulcer (ulcer on the forearm: whole forearm; ulcer on the upper arm: whole upper arm; ulcer on the lower leg: whole lower leg; ulcer on the thigh: whole thigh). On top of the PCM-bandage a layer of heat insulation material will be placed and fixed with an elastic bandage. This PCM-bandage will be renewed 12 hourly to guarantee a skin temperature 40°C over the whole treatment period.
3. A small portable data-logger will be connected to the temperature sensor and will automatically measure and store the temperature every 10 minutes over the whole period of treatment.

**Adverse events (risk assessment)**

Risk of secondary infection of the wound (ulcer)

No problem is foreseen with the following procedure: The wound will daily be cleaned with a desinfective and covered with polyvidon paste, sterile pads and a bandage

Risk of burning of the skin

The temperature a PCM attains during the crystallization process is defined by the thermo-physical properties of the substance selected. This crystallization temperature cannot by any means be surpassed during the course of the treatment. Since the crystallization temperature of the PCM selected for our therapy is significantly below the temperature where skin damage can occur heat trauma is ruled out.

**Stopping rules**

At weekly intervals it will be decided if a patients needs to be taken out of the study cohort due to worsening of the ulcer or arrested healing. The following scenarios with the corresponding sets of criteria listed will be used:

(1) improved (criteria: evidence of progressive ephithelialization and / or scaring and / or collapse of undermined edges)

(2) unchanged (criteria: no ephithelialization and no scaring and no collapse of undermined edges

(3) worsened (criteria: increase in size of the lesion).

A patient will be offered the standard national surgical treatment protocol if the lesion worsened between two assessments 1 week apart (scenario c) or remained unchanged between two assessments 3 weeks apart (scenario b).

Excised tissue will be analyzed by immunohistology to assess mycobacterial tissue burden, signs of granulomatous responses and granulation. Surgical specimens from patients receiving conventional treatment will serve as controls.

The heat treatment will be applied for 4 weeks if stopping rules do not apply. If the ulcer is not completely healed two months after the treatment period of four weeks the patient will be offered surgical treatment following national guidelines. The same will be offered directly after 4 weeks of heat treatment in case a punch biopsy from day 21 shows high bacterial load.

For subgroup analysis the following samples will be collected and analysed: Blood samples (10 ml) for analysing humoral and cellular immune responses will be taken on days 0, 14, 30 and 60 from enrolled patients. Samples from patients receiving conventional therapy and from community controls will be included in the analysis.

**Data recording (CRFs see annex)**

1. The temperature at the skin surface (channel 2 of the data logger) and the ambient temperature (channel 1 of the data logger) will be automatically recorded every 10 minutes over the whole treatment period of 4 weeks. At regular intervals the temperature data will be transferred to a notebook to conduct interim analyses of the skin temperature curve and to save a back-up data set in case of a data logger failure. Additionally, the skin and ambient temperature will be read and recorded at the times of renewal of the PCM-bandage (12-hourly).
2. Daily the ulcer will be measured and photographed with an electronic camera with a scale in place at the time of redressing of the wound. The edge of the ulcer will be assessed with respect to collapse of undercutting edges, epithelialization and development of scar tissue (recorded in mm from edge of the ulcer at four points (opposite sites on max. / min. diameters). The photographs will be stored on the notebook.

**Follow-up**

For all those who have completed the heat treatment successfully the follow-up is for 6 months to assess cure and for 18 months to observe recurrences (clinical endpoints). During the first follow-up phase the visits are monthly (more frequently, if necessary). During the second phase the follow-up visits are 3 monthly (more frequently, if necessary). The data collected at the follow-up visits will be recorded on the form “Follow-up visit form” (see CRFs).

**Recurrent lesions**

Recurrent lesions will be offered the standard national surgical treatment.

**Data storage**

The data collected during the pilot trial will be recorded on forms at the bedsite of the patient during enrolment (see CRFs “Demographic information”, “Examination on enrolment”) and twice daily during treatment (see CRF “Daily record form”).

Of all patients who have completed the pilot trial successfully the data collected at the follow-up visits will be recorded on the form “Follow-up visit form” (see CRFs).

The laboratory data will be entered by Partner 3 at the Swiss Tropical Institute in Basel (see CRF “Laboratory record form”).

All data will be entered into a data base (Epidata) on a notebook during the trial. The data recorded during the trial in Ayos and the laboratory data recorded in Basel will be joint into one data base (Epidata) by Partner 1 and 2 and the completely anonymized data set will be handed over to all partners to carry out their part of the analysis.

**Data analysis**

Specific data will be analyzed by the individual groups according to their expertise: clinical trial data (Partner 1 and 2), microbiological, molecular and immunological data (Partner 3) and thermal data (Partner 4). The final overall analysis of all 3 data sets will be done by all partners together.

Main outcome variables are (a) completing 4 weeks of heat treatment and (b) cure 6 months after completing 4 weeks of heat treatment and (c) recurrence rates after 6 and 18 months after completing 4 weeks of heat treatment.

Secondary outcome variables are tolerability of the heat treatment and immunological responses of patients during and after the heat treatment compared to controls and surgically treated patients.

**Environmental impact**

No negative environmental impact of the research activities is discernible.

**Ethic considerations**

The Ethics Committees of the University Hospital Heidelberg and the national ethical committee of Cameroon approve in writing the conduct of this clinical study, together with the investigator’s informed consent document, prior to study initiation.

In performing this study, both the Investigator and sponsor endorse, as a minimum, the standards for conduct of clinical research activities as set forth in the Declaration of Helsinki and local country laws and regulations.

**Informed consent (see consent forms in the annex)**

The Investigator will obtain written informed consent from each subject enrolled in the study, in accordance with the current version of the Declaration of Helsinki and the laws and regulations of Cameroon where the investigation is being conducted.

It is the responsibility of the Investigator to assure that the patient (or guardian or legal representative) has signed the Informed Consent before any activity or treatment is undertaken which is not part of routine care.

**Confidentiality**

Only source documents, which remain with the Investigator, and participants’ logs established by the Investigator will contain the participants’ personally identifiable information. No names or other identifiers of the subjects will ever be recorded or reported in association with the study data.

Reports of the research project will not include any names or any personal information that could reasonably be associated with any research participant.

Electronic databases will be password-protected, and will only be accessible by study personnel.

The data containing patients names or other identifiers will be destroyed after 5 years.

**Time schedule**

***November 2005***

First visit of the trial site (A. Um Book, M. Hellmann, G. Pluschke, T. Junghanss) to

1. introduce the treatment modality and the trial to the hospital team
2. assess the performance of the device (PCM-bandage and temperature monitoring) on site
3. to adapt the trial protocol

***December 2005 – February 2007***

1. Ethical clearance through the respective bodies in Cameroon and Germany
2. Final design of the PCM-bandage
3. Performance checks of the bandage, particularly with respect to the heat storage and temperature requirements
4. Second visit of study team in Ayos (December 2006)
5. Transport of the equipment to Ayos
6. Active case finding in the district

***1- 20 February 2007***

Preparation of the trial side

***21 – 26 February 2007***

Enrolment of patients until 10 patients are selected

***21 February – 26 March 2007***

Treatment and data monitoring of 10 patients until all patients for which stopping

rules did not apply have received 4 weeks of therapy

***27 March – 30 March 2007***

Preparing for long term follow-up of treated patients and jointly concluding and informing the community

***26 March – mid April 2007***

Preliminary data analysis and feed-back to Ayos hospital staff

***Mid April – mid November 2007***

Monthly follow up of patients (to assess cure)

***Mid November 2007 – mid December 2007***

Preliminary data analysis and feed-back to Ayos hospital staff

***Mid December 2007 – mid 2008***

3-monthly follow-up to identify recurrences

***July 2008***

1. Final data analysis and feed-back to Ayos hospital staff
2. Publication of results

**Protocol amendments at enrolment before start of treatment (28.2.2007)**

We reduced the total number of patients enrolled into the study from 10 to 7, extended the age range from 8 – 30 years to 6 - 30 years and made no restrictions with regard to the lower limits of the diameter of ulcers. This allowed us to recruit all patients within 4 days and to highly synchronize the study activities. We thereby adapted to the local situation at the time of the trial and achieved conditions which guaranteed high trial standards. We renewed the PCM-bandage 8-hourly to increase safety with regard to possible adverse reactions and not 12-hourly as originally planned in the protocol.
